# Role of dexmedetomidine infusion after coronary artery bypass grafting

**DOI:** 10.1186/s43057-019-0014-8

**Published:** 2020-01-22

**Authors:** Tamer Hamouda, Mohamed Ismail, Tamer Hamed Ibrahim, Hesham Ewila, Ahmed Elmahrouk

**Affiliations:** 1 Cardiovascular Department, King Faisal Specialist Hospital and Research Center, Jeddah, Saudi Arabia; 2 Cardiothoracic Surgery Department, Faculty of Medicine, Benha University, Benha, Egypt; 3 Cardiothoracic Surgery Department, Faculty of Medicine, Mansoura University, Mansoura, Egypt; 4 Department of Anaesthesia, Faculty of Medicine, Ain Shams University, Cairo, Egypt; 5 Department of Anaesthesia, Faculty of Medicine, Suez Canal University, Ismailia, Egypt; 6 Cardiothoracic Surgery Department, Faculty of Medicine, Tanta University, Tanta, Egypt

**Keywords:** Dexmedetomidine, Coronary artery bypass grafting, Postoperative pain, Alpha 2 agonists

## Abstract

**Background:**

Postoperative pain has negative consequences on patients’ outcomes after cardiac surgery. Routine management with opioid and or non-steroidal anti-inflammatory medications has several disadvantages. Dexmedetomidine is a selective α2 agonist used for sedation and analgesia. The use of dexmedetomidine for postoperative pain management and decreasing delirium and agitation in cardiac surgery patients is a matter of debate. Our objective was to determine the role of an early administration of dexmedetomidine in decreasing opioid use post-cardiac surgery and its effects on the quality of postoperative recovery.

**Results:**

Medical records of 120 patients admitted to the cardiac surgery intensive care unit (CSICU) after coronary artery bypass grafting (CABG) in two cardiac centers between December 2015 and December 2016 were reviewed. Patients were divided into two groups. Group A included 55 patients who received dexmedetomidine in a dose of 0.2–0.4 mcg/kg/h on admission to CSICU, and group B included 65 patients who did not receive dexmedetomidine. The primary outcome was the pain score immediately after extubation, and the secondary outcomes included post-extubation sedation and pain scores for 12 h.

There were significant decrease of the pain scores in dexmedetomidine group that continues through the 3rd, 6th, 8th, and 12th hour readings after surgery with mean modified Ramsay scores 0.1 ± 0.0, 0.89 ± 2.05, 0.35 ± 0.1, and 0.12 ± 1.1 respectively compared to 0.46 ± 1.15, 3.46 ± 2.93, 0.98 ± 1.90, and 0.12 ± 1.1 in group B (*p* < 0.001), significant decrease in cumulative morphine received (*p* < 0.001, OR = 909, 95% CI 0.05–0.19), favorable reduction in heart rate in dexmedetomidine group (80 ± 1.9 b/min) compared to 96 ± 8.8 b/min in the other group (*p* = 0.017), and smoother recovery from general anesthesia.

**Conclusion:**

Administration of dexmedetomidine in the early postoperative period can be safe. It may reduce the use of opioids, has sedative, analgesic, and sympatholytic effects that could play a useful role during the management of coronary artery bypass patients, and may improve postoperative recovery.

## Background

Postoperative pain after cardiac surgery has a negative impact on the outcome, in addition to the patients’ experience. Tachycardia, hypertension, arrhythmias, and myocardial ischemia are frequent postoperative findings. Most postoperative pain management protocols are opioid-based, which have a dose-dependent analgesic effect [1].

On the other hand, opioids have a number of undesired side effects, including nausea, vomiting, decreased gastrointestinal motility, respiratory depression, drowsiness, and hemodynamic effect with large doses [2]. Moreover, non-opioids analgesics, such as non-steroidal anti-inflammatory drugs and acetaminophen, may be a useful complement to opioids for postoperative pain relief. Additionally, non-opioid analgesics can reduce the need for opioids and minimize its side effects. However, the efficiency of these medications can be limited, as it can seriously affect the patients postoperatively because of their effect on bleeding and renal function [3].

Dexmedetomidine (DEX) is a highly selective α2 receptor agonist that provides better sedation, analgesic, and anxiolytic effect. This property is considered unique among sedatives used for intensive care units generally [4, 5]. Many studies on DEX in postoperative high-risk non-cardiac patients showed that those patients required significantly lower sedative agents. Furthermore, dexmedetomidine has a less hemodynamic effect and non-significant respiratory depression [6, 7]. Additionally, patients who received DEX experienced mutual sedation and smooth extubation, and they had lower mortality [7].

Moreover, recent findings show that such patients have a lower incidence of delirium over comparators [8, 9]. On the other hand, other studies argued the effect of DEX in decreasing the incidence of postoperative delirium, especially in older patients [10, 11]. In this retrospective study, we tried to examine the efficacy of post-coronary artery bypass grafting (CABG) DEX infusion in reducing postoperative morphine consumption and in improving the quality of postoperative recovery.

## Methods

### Design and patients

Data of all patients admitted to the cardiac surgery intensive care unit (CSICU) following CABG, between December 2015 and 2016, were retrieved from the medical records after approval from the local medical ethics committee. Patients’ confidentiality was maintained by removing any identifying information from the data set before further usage and analysis. The variables and their values were coded into an alphanumeric format for concealment with few designated persons having the coding key.

A hundred and twenty patients were included in the study. Patients with renal failure, prolonged postoperative intubation for more than 8 h because of hemodynamic instability or bleeding, and patients who had re-exploration were excluded.

Patients were divided into two groups: group A included 54 patients who received postoperative DEX infusion for sedation, and group B included 66 patients who did not receive DEX.

### Study protocol and outcomes

All patients were intubated, ventilated, and sedated by propofol intravenous (IV) infusion 25–50 μg/kg/min. On admission to CSICU, vital signs were recorded, and group A patients were started on DEX IV infusion 0.2–0.4 μg/kg/h. while patients in group B were maintained only on propofol infusion.

Vital signs, including heart rate (HR), oxygen saturation, and end-tidal carbon dioxide, were continuously monitored, and blood pressure was monitored both invasively and non-invasively. Patients were started on acetaminophen 1 g IV every 6 h, and 2–3 mg of morphine were administered intravenously if the pain was suspected in case of unexplained tachycardia and hypertension when the patients were still intubated.

We reviewed the difference in total morphine consumption and vital data between the two groups while patients were intubated. Our protocol was to start weaning the patients from sedation and prepare for extubation 2 h after admission to CSICU and after ensuring the stable hemodynamic and full return of muscle power, so once these criteria were met, propofol and DEX infusion were turned off, and patients were extubated after full recovery.

Our primary outcome was the pain score assessed by the primary nurse and physician immediately after extubation. The pain was evaluated according to the numeric pain intensity scale (NPIS), ranging from no pain (0) to severe non-tolerable pain (10).

We reviewed the pain scores and vital signs every hour in all patients for the first 12 h post-extubation in addition to sedation score which was assessed by modified Ramsay score (MRS) and ranged from 1 to 6 as follows: 1 = anxious/agitated, 2 = cooperative oriented, 3 = responds to commands only, 4 = asleep with brisk response to light glabellar tap (LGT), 5 = sluggish response to (LGT), 6 = asleep no response to (LGT).

Our protocol was to administer 2–3 mg of morphine to the patient once the pain score is ≥2, and the total morphine consumption in the first 12 h post-extubation in both groups was reviewed, and that was our secondary outcome in addition to the vital data and sedation scores in the first 12 h after extubation.

### Statistical analysis

Statistical analysis was performed using SPSS 15 for Windows (IBM Corp, Chicago, IL, USA). Continuous data were presented as mean ± standard deviation and categorical data as count and percent. *T* test was used to compare the continuous variables, and chi-squared test or fisher exact test was used for categorical variables when appropriate. The trend of pain severity was investigated by a repeated measure analysis of variance (ANOVA) model. Freedom from the first dose of morphine was plotted using the Kaplan-Meier survival curve and compared between both groups using a log-rank test.

## Results

One hundred and twenty patients with a mean age of 56 ± 10.1 years were included in the study. The mean left ventricular ejection fraction (LVEF) was 40 ± 9.4% in the DEX group compared to 44 ± 7.3% in group B (*p* = 0.75). The average time of anesthesia was 312 ± 66 min in group (A) and 309 ± 42 min in group (B) (*p* = 0.54). Patients stayed in the intensive care unit (ICU) after surgery with a mean time of 2.9 ± 1.1 days and a range of 2 to 4 days.

Fifty-four patients had intravenous DEX started on admission to the intensive care unit, and 66 patients were started on propofol. Analysis of baseline variables of the patients was presented in Table 1, which showed no significant differences between the two groups.

**Table 1:** Comparison of the preoperative, operative, and postoperative data between groups (continuous data are presented as mean and standard deviation or median and categorical data as number and percent)

	Group A (*n* = 54)	Group B (*n* = 66)	*p* value
Age (years)	54 ± 9.5	57 ± 8.3	0.181
Weight (kg)	85 ± 10	80 ± 9.3	0.530
Height (cm)	1.61 ± 9	165 ± 3	1.511
Ejection fraction	40 ± 9.4	44 ± 7.3	0.752
Duration of anesthesia (min)	312 ± 66	309 ± 42	0.541
Operative time (min)	248 ± 18	251 ± 92	0.572
Bypass time (min)	110 ± 38.5	105 ± 40.3	0.641
ICU stay/days	2 ± 0.5	3 ± 0.1	0.512
Intubation time (hours)	6 ± 2.3	8 ± 7.9	0.231
MAP (mmHg)	77.93 ± 5.9	74.53 ± 8.3	0.561
Heart rate	80 ± 1.9	96 ± 8.8	0.017
Modified RS (median)	2	3	0.802
Pain intensity score median	1	2	0.113

*cm* centimeters, *min* minutes, *ICU* intensive care unit, *MAP* mean arterial pressure, *HR* heart rate, *Modified RS* modified Ramsay score

The severity of pain was measured by 10 points in NPIS in different time intervals: 1st, 3rd, 6th, 12th, 18th, and 24th hours (Table 2).

**Table 2:** Pain score in different time interval after cardiac surgery (data are presented as mean and standard deviation)

Group	1st hour	3rd hour	6th hour	8th hour	12th hour	24th hour
Group A (*n* = 54)	0.39 ± 1.25	0.1 ± 0.0	0.89 ± 2.05	0.35 ± 0.1	0.12 ± 1.1	0.0 ± 0.90
Group B (*n* = 66)	1.08 ± 0.67	0.46 ± 1.15	3.46 ± 2.93	0.98 ± 1.90	0.30 ± 0.2	0.0 ± 0.96

Patients experienced a period of analgesia after surgery. The most severe pain had a point that was less than 3. By the 6th hour after surgery, the pain started to appear gradually and became more severe. The severity of pain diminished until the disappearance at the 18th hour after surgery (*p* < 0.001).

**Fig. 1:**
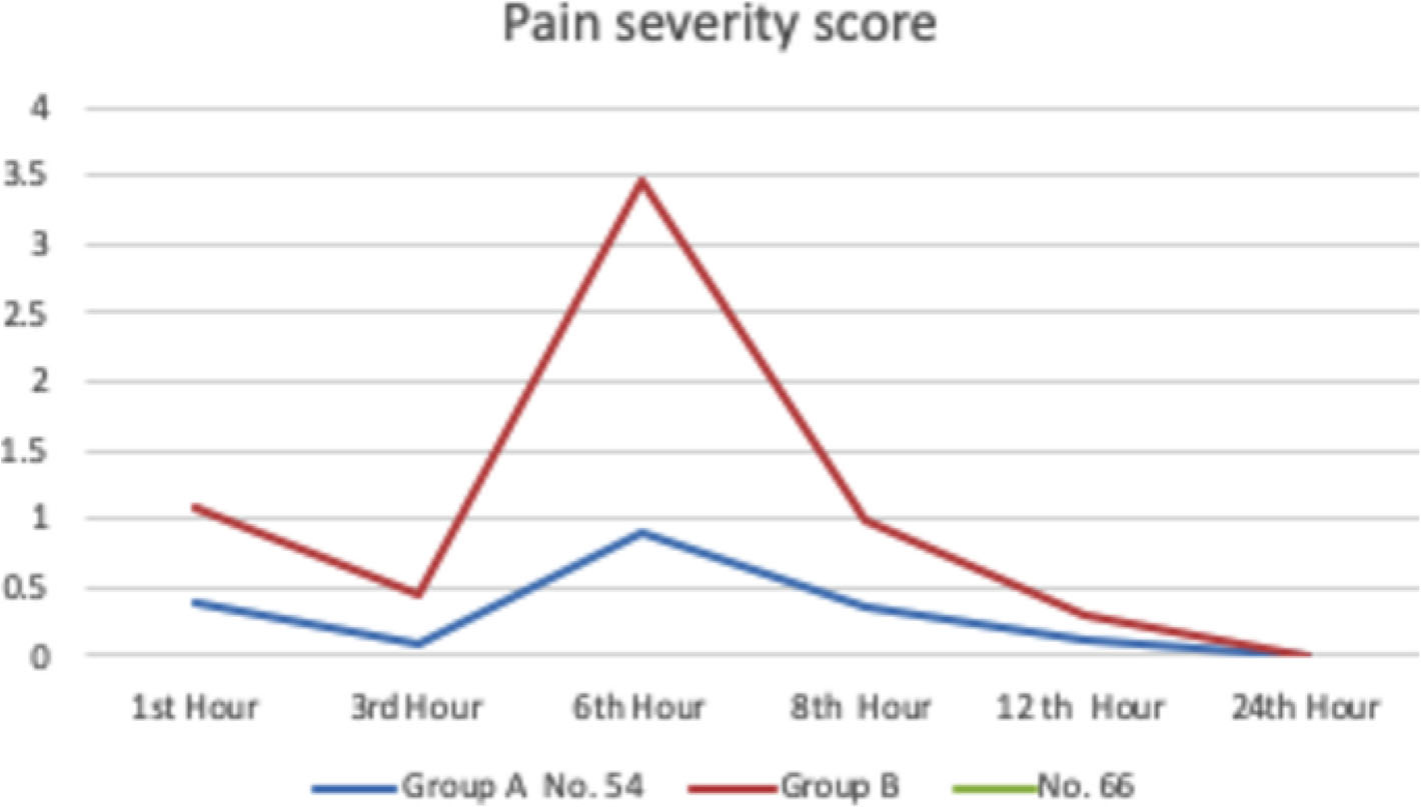
Changes of pain severity score in study groups

In the first hour after the operation, the trend of pain severity was equal in both groups. The effect of analgesia continues in those who received dexmedetomidine until the 3rd hour after surgery, while in the control group, the pain severity was increasing. The patients in the DEX group had statistically significant lower pain severity compared to the control group throughout the first postoperative day (*p* value < 0.001) (Fig. 1).

The assessment was done every hour during intubation and 2-hourly after extubation; the mean sedation score was analyzed all through intubation (Table 3). We observed that intubation time was 6 ± 2.3 h in the DEX group compared to group B with 8 ± 7.9 h (*p* = 0.231). The mean arterial pressure had increased in both groups within the first 1 h and dropped within the first 2 h then became stable in the DEX group with a lower heart rate than the other group.

**Table 3:** Sedation score during intubation and after extubation (data are presented as the mean)

	During intubation	Immediately after extubation	2 h after extubation	4 h after extubation	6 h after extubation	8 h after extubation
Group A (*n* = 54)	5.2	3.2	2.9	2.7	2.2	2.3
Group B (*n* = 66)	4.5	2.1	2.1	2	2.1	2.1

In the DEX group, 10% of patients needed to receive morphine for pain compared to 95% of the patients in the other group (*p* < 0.001, OR = 909, 95% CI 0.05–0.19). Moreover, the dose of received morphine sulfate was lower in the DEX group (1.0 ± 1.2 mg) compared to 2.9 ± 1.8 mg in the other group (*p* < 0.001).

**Fig. 2:**
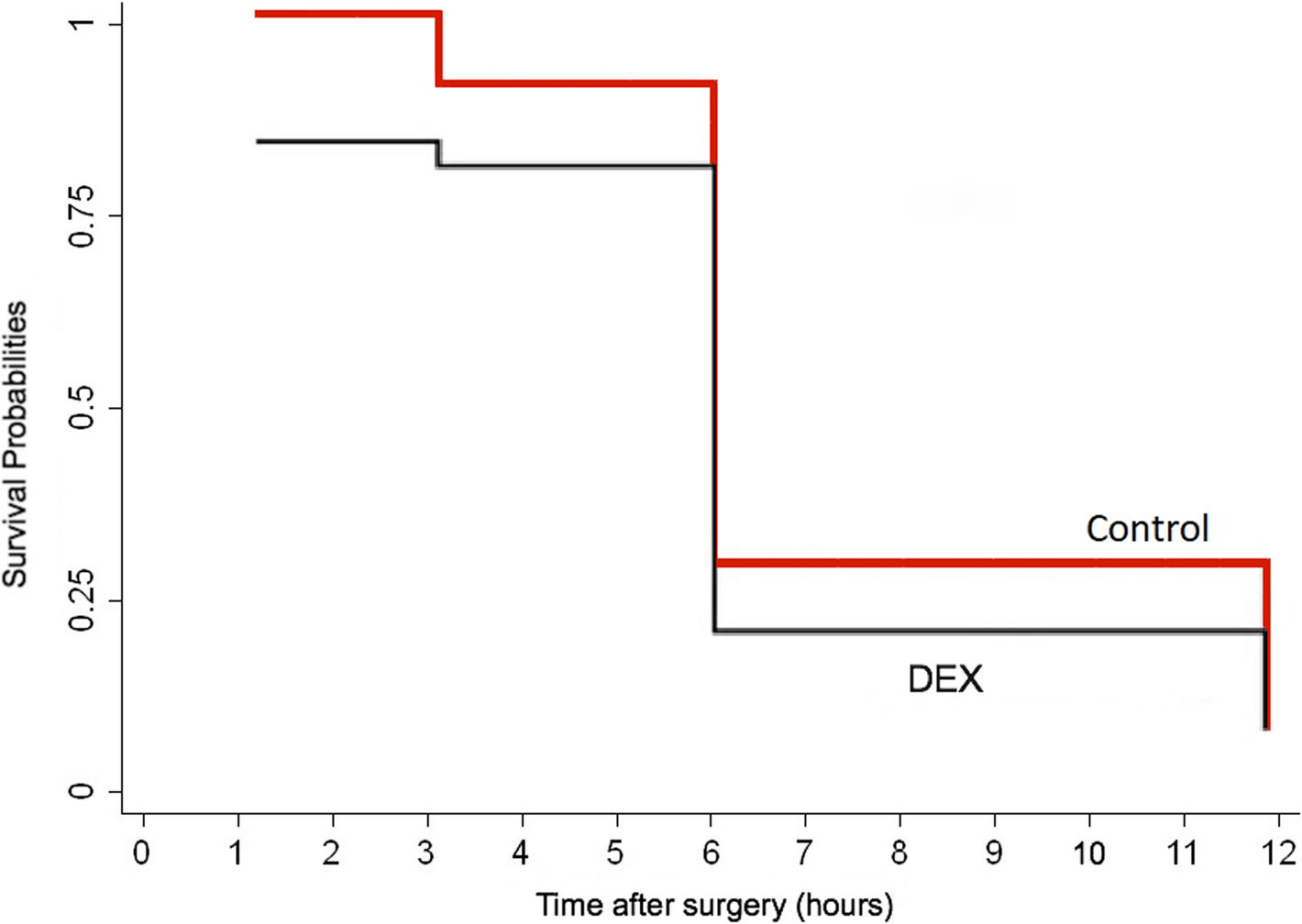
Kaplan-Meier curve for estimation of the time to receive the morphine

The time of prescription of the 1st dose of morphine sulfate could be considered as a proxy for the beginning of intolerable pain (pain score between 4 and 5) (Fig. 2). The pain became relatively unbearable 6 h after finishing the surgery. No significant difference between the two groups was noticed using the log-rank test (*p* = 0.09). The pattern of receiving the first dose of morphine sulfate was similar in both groups.

## Discussions

This study demonstrated that early infusion of DEX following CABG operations was associated with a reduction of morphine usage, a decrease in pain severity, and improvement of sedation, which encouraged early extubation. Pain control is crucial after cardiac surgery. The release of catecholamine causes the increase of the peripheral vascular resistance, heart rate, and risk of myocardial infarction, arrhythmias, and mortality. From our observations, we noticed a transient rise in main arterial pressure (MAP) during the 1st postoperative hour in both groups, followed by a decrease in MAP.

Arain and associates [12] found a brief increase in blood pressure in the DEX group, which was related to activation of α2 agonist on the smooth muscle of the vessel wall, which leads to transient vasoconstriction and increased in mean arterial pressure. We have noticed this in both groups. However, we thought these initial hemodynamic changes in MAP and HR were most probably related to post-surgical stress, the surgical procedures itself, or pre-existing hypertension as it happened in both groups [13]. The decrease in MAP and HR was related to more gradual central effect of DEX in patients who received it, while the reduction of MAP for patients who did not receive DEX and depended on morphine as main analgesic most probably was related to decreasing catecholamine and direct vasodilatation effect of morphine considering that there was no significant difference in the number of patients receiving inotropes and vasodilators between the two groups.

Our findings were in line with Liu and coworkers’ [14] meta-analysis, which stated that DEX lead to a shorter length of intubation, but it can be associated with bradycardia in patients after cardiac surgery compared with propofol. This tendency to lower the heart rate and systolic blood pressure with decreased incidence of tachycardia and arrhythmias have a cardioprotective effect, as stated by another meta-analysis by Gong and coauthors [15].

On the other hand, Mukhtar and colleagues [16] concluded in their study that intraoperative DEX infusion attenuated the hemodynamic and neuroendocrine response to surgical trauma and cardiopulmonary bypass. The same findings were declared by Priye and associates [17], who concluded that DEX infusion, even those without the loading dose, have a safe, effective adjuvant analgesic effect. It can reduce narcotic consumption without undesirable hemodynamic effects in cardiac surgery patients.

Shehabi and coworkers [11] found that DEX reduced vasopressors’ requirement after cardiac surgery with effective analgesia sedation, less hypertension, and more bradycardia versus morphine regimen. In our study, we did not find any difference between the two groups with regard to MAP and HR (*p* = 0.561 and 0.017, respectively). Barletta and associates [18] stated that DEX decreased cardiac index and HR for patients undergoing lengthy procedures which remained reduced at the time of discharge; our results did not support this conclusion as there was no significant difference between the two groups in HR. On the other hand, Maldonado and coworkers [19] concluded that DEX was associated with a lower rate of respiratory depression. It caused a higher rate of adverse hemodynamic events, which might be a concern in a hemodynamically unstable patient, but we did not observe any adverse effect on hemodynamic stability; this can be attributed to the low number of patients who needed inotropic support or vasodilator during the post-operative period in our cohort. Moreover, we have estimated opioid administration as analgesics postoperatively, which could reflect passively on the patients’ hemodynamics post-CABG. We observed that 10% of the patients were in need of opioids in the DEX group. These patients could be considered to have a low pain threshold or the post-operative managing doctors had a low threshold to give his patients more analgesia.

It worth mentioning that, while 95% of the control group were depended on opioids, 5% only of them only received non-steroidal anti-inflammatory (NSAID), according to the assessment of the post-operative ICU doctors who considered most of these patients with high pain threshold, and so, NSAID was enough to control their pain. Moreover, the dose of opioids given in the DEX group was significantly less than those of the other group [20, 21].

The pattern of pain in both groups was almost the same, which means that DEX does not alter the trend of the pain but decreased the intensity, and both groups needed to receive analgesics at the same point according to pain score between 4 and 5. These results agreed with Cheng and associates’ study [22].

Some authors mentioned that the use of DEX decreased the length of ICU stay in comparison with the use of other sedative and analgesics like midazolam and morphine, and we did not observe in our study significant differences in ICU stay as it was 2 ± 0.5 and 3 ± 0.1 days (*p* = 0.512). There are many factors other than pain and analgesics which could influence the ICU stay, including the patient’s hemodynamics, which can be affected by other etiologies other than pain.

From the clinical point of view, Lin and coworkers [9] suggested that DEX gave a suitable condition through which it facilitated weaning process from mechanical ventilator as it did not depress spontaneous ventilation, as well as it decreased the risk of delirium, ventricular tachycardia, and hyperglycemia following cardiac surgery; however, it could cause significant bradycardia. In our study, there was no significant difference between the two groups in intubation time.

### Study limitations

The primary limitation of the study is the retrospective nature, and several factors may have affected the outcomes other than the treatment. A randomized clinical trial is required to adjust for the measured and unmeasured variables that could confound the results. However, because the administration of DEX in our cohort was not related to patients’ specific risk factors, the two groups had comparable baseline data.

## Conclusion

Administration of dexmedetomidine in the early postoperative period can be safe. It may reduce the use of opioids, has sedative, analgesics, and sympatholytic effects that could play a useful role during the management of coronary artery bypass patients, and may improve postoperative recovery.

## Data Availability

Data are available with the corresponding author upon request.

## Abbreviations

CABG: Coronary artery bypass grafting
CSICU: Cardiac surgery intensive care unit
DEX: Dexmedetomidine
HR: Heart rate
ICU: Intensive care unit
LGT: Light glabellar tap
LVEF: Left ventricular ejection fraction
MAP: Main arterial pressure
MRS: Modified Ramsay score
NPIS: The numeric pain intensity scale
NSAID: Non-steroidal anti-inflammatory
